# Identification of key gene signatures for the overall survival of ovarian cancer

**DOI:** 10.1186/s13048-022-00942-0

**Published:** 2022-01-20

**Authors:** Akash Pawar, Oindrila Roy Chowdhury, Ruby Chauhan, Sanjay Talole, Atanu Bhattacharjee

**Affiliations:** 1 Section of Biostatistics, Center for Cancer Epidemiology, Tata Memorial Centre, Mumbai, India; 2 Homi Bhabha National Institute, Mumbai, India

**Keywords:** Ovarian cancer, Integrative analysis, Clinical prediction, Gene signature, Data visualization

## Abstract

**Background:**

The five-year overall survival (OS) of advanced-stage ovarian cancer remains nearly 25-35%, although several treatment strategies have evolved to get better outcomes. A considerable amount of heterogeneity and complexity has been seen in ovarian cancer. This study aimed to establish gene signatures that can be used in better prognosis through risk prediction outcome for the survival of ovarian cancer patients. Different studies’ heterogeneity into a single platform is presented to explore the penetrating genes for poor or better survival. The integrative analysis of multiple data sets was done to determine the genes that influence poor or better survival. A total of 6 independent data sets was considered. The Cox Proportional Hazard model was used to obtain significant genes that had an impact on ovarian cancer patients. The gene signatures were prepared by splitting the over-expressed and under-expressed genes parallelly by the variable selection technique. The data visualisation techniques were prepared to predict the overall survival, and it could support the therapeutic regime.

**Results:**

We preferred to select 20 genes in each data set as upregulated and downregulated. Irrespective of the selection of multiple genes, not even a single gene was found common among data sets for the survival of ovarian cancer patients. However, the same analytical approach adopted. The chord plot was presented to make a comprehensive understanding of the outcome.

**Conclusions:**

This study helps us to understand the results obtained from different studies. It shows the impact of the heterogeneity from one study to another. It shows the requirement of integrated studies to make a holistic view of the gene signature for ovarian cancer survival.

## Background

Several treatment strategies evolved to make the outcome of ovarian cancer better [1]. But the five-year overall survival (OS) of the advanced stage of the disease remains nearly 25-35% [2]. A considerable amount of heterogeneity and complexity has been seen in ovarian cancer (OC) [3]. Genetic alterations (BRCA gene mutations, DNA damage, TP53 mutations, chromosomal instability) and alterations in RNA and miRNA expression characterised the high grade serous ovarian cancer (HGSOC) [4]. Simultaneously, genes such as TRIM44 and CENPK [5] were identified, and these were significantly associated with the prognosis of ovarian cancer patients. However, only a few prognostic signatures have been developed [5]. Hence, there was a need to study the disparities among different studies and accumulate that in a single platform to understand the penetrating genes for poor or better survival of ovarian cancer patients.

The predicting tool needs to be robust and flexible to accommodate gene signature and provides treatment outcomes. Individual-level risk prediction score generated by the rigorous statistical model always served the purpose. It was not only about the model development; moreover, the statistical model compatible with suitable data fulfilled the mission. Omics data only support individual-level risk score prediction or personalised medicine by coupling with survival outcomes. So the survival outcome and omic data come together to support the personalised medicine and generate the individual-level risk score. Omic data were having several thousand gene expression defined as high dimensional data. The joint work of survival data and high dimensional data is not new [6]. There are several challenges in high-dimensional data [7], where many of the problems were solved, and many of them were not. Separately, survival analysis challenged with different methodology [8].

Mostly oncology domain presented with follow-up observations like recurrence, response and death. The time-to-occurrence of the event plays a crucial role in this direction.

This work is prepared by applying the risk prediction model in the survival - high dimensional ovarian cancer data.

The Cox proportional hazards (PH) regression model is defined as

1 $$h(t)={h}_0(t)\mathit{\exp}(x)$$

where *h*(*t*) is defined as the hazard function, (t) appear for the survival time, X is the covariate vector, the coefficient β measures the impact which is effect size and the *h*_0_(*t*) is known as the baseline hazard [9].

When there are multiple coefficients and covariates,

2 $$h(t)={h}_0(t)\mathit{\exp}\left({\beta}_1{x}_1+{\beta}_2{x}_2+{\beta}_3{x}_3+.\dots +{\beta}_n{x}_n\right)$$

the quantities of *exp*(*β*) are called as the hazard ratios, and here a value of *β*_*i*_ greater than zero or the hazard ratio greater than one depicts that the value of the i^th^ covariate will increase and overall the event hazard will increase and thus the survival length will decrease.

It was anticipated that while the initial treatment was given, a patient may be no longer censored. The dimension reduction was the common challenge to work with high dimensional data, and it was not easy to obtain any unique, robust estimator [10]. The dimensional reduction has been extended in this line as the linear models [11]. The most widely used methods are linear and ridge regression model. By reducing the dimension, it becomes compatible with a penalised estimator of the Lasso. However, it was not stable for a large number of variables in microarray data [12]. The survival modelling with high-dimensional covariates becomes complicated. It required to look first about clustering the gene expression data and looks at clustered data by the Cox PH model.

Recently gene signatures have been commonly adopted for cancer patients. It helps to determine the best therapy in the context of personalised medicine [13], and it was confirmed by the clinically validating procedure [14]. Over the years, the selection of gene signature and further adoption in cancer prediction took a lead role in cancer research. However, it was challenging to identify the reliable gene signature due to variable selection challenges. Commonly the selected gene signature was found inconsistent from study to study [15]. Because the model used to select gene signature from one study also varies from others [15]. It raised the contradictory outcomes between the studies [13]. This signature was concerned with the reliability and benefits of using it as reliable for clinical practice.

Several environmental factors may influence the study. Given the relatively small sample size, conclusions were preliminary lacked due to power and generated with less accuracy of prediction [15]. Sometimes, the prevalence of the clinical outcome also reduced the replicability [16].

Recent studies showed that due to the low frequencies of some molecular types become inappropriate to predict for the new patients correctly. It would be worth testing how well the method performs in different laboratories. The laboratory wise variation in gene signature accuracy prediction was observed.

Data processing technique also play a crucial role to obtain different outcomes. Particularly, the normalization steps for data processing was crucial [16].

It required to create of the training data set before validation. Perhaps, result bias also generated due to the small sample size.

It could happen in some situations and thus requires a more detailed look on the microarray chip [17].

It becomes challenging to maintain consistency if the new study performs into a new platform to boost up the prediction capacity. The gene expression measured by next-generation sequencing was different “from microarray measurements. It needs to derive a methodology from boosting up the clinical prediction capacity for a specific platform.

Different statistical methods have been developed to integrate data from different studies towards agreement on a conclusion where the horizontal integration was useful for a conclusive remark [18].

This study aimed to establish a gene signature which can be helpful in better prognosis through risk prediction model in the high dimensional-survival ovarian cancer data obtained from the NCBI’s Gene Expression Omnibus database (https://www.ncbi.nlm.nih.gov.in/geo/).

## Methods

A total of 6 high dimensional-survival ovarian cancer data sets was obtained to understand the gene expression and the process followed by them, to conclude for better prognosis through a risk prediction model. An integrative approach of multiple data sets was made to find out the gene to make the influence of poor/better survival by considering different data sets. The Cox PH model was used to obtain significant genes that had an impact on ovarian cancer patients, emphasising the importance of the effect size. The gene signatures were prepared by splitting over-expressed and under-expressed genes together after the gene selection. The chord plot data visualisation technique was used to formalise the recommendations for routine clinical practice. The data visualisation techniques were prepared to predict the overall survival, and it could support the therapeutic regime.

### Identification of differentially expressed genes in ovarian Cancer

In this study, data was retrieved about gene expression from studies conducted on the NCBI’s Gene Expression Omnibus database (https://www.ncbi.nlm.nih.gov.in/geo/) to identify relevant data sets which contain the gene profile of ovarian cancer. Data sets with accession number GSE14764, 22283 probes ID of 80 patients [19]; accession number GSE17260, 41000 probes ID of 110 patients [20]; accession number GSE19829, 12558 probes ID of 42 patients [21]; accession number GSE30161, 54613 probes ID of 58 patients [22]; accession number GSE49997, 32878 probes ID of 194 patients [23] and accession number GSE63885, 54613 probes ID of 75 patients [24] were chosen for the study.

### Genes selection

The data sets were filtered with *p*-value < 0.05 by univariate Cox PH model. A number of probes were selected, and the VIMP function in the ‘party’ package in R was used to obtain the most significant probes. The correlated genes were separated by VIMP function in R. The hazard ratio (HR), and confidence intervals were considered to select the probes. Later, the positive probes having HR > 1 and negative probes having HR < 1 were selected to create the adjacency matrix. The adjacency matrix was generated from the positive and negative probes, which was used to create the chord plot (a circular visualisation to show relations between genes by links).

Data set obtained from the Gene Expression Omnibus (GEO) database under the accession number GSE14764. It consisted of 22,283 probes of 80 patients. Initially, the data set was filtered with *p*-value < 0.05 by univariate Cox PH model, where a total of 531 probes were selected. The VIMP function obtained the most significant 100 probes. The correlated genes were separated by the VIMP function. The HR and confidence intervals were considered to select 100 probes.

In the next step, the positive probes having HR > 1 and negative probes having HR < 1 were selected for the adjacency matrix. The adjacency matrix of 10 *10 was generated from the positive and negative probes, which was used to create the chord plot. A similar approach was used for all the data sets.

### Generating global *p*-value

Suppose n number of univariate statistical tests were performed. The statistics obtained from each step were presented as *X*_*i*_. Now, n number of the statistical test will generate *X*_1_, *X*_2, ……_*X*_*n*_. The statistics was presented as vector $\overline{X}=\left({X}_1,{X}_{2,\dots \dots }{X}_n\right)$. The statistics obtained from each test followed certain distribution as $\overline{X}$ presented a random variable. The *P*-value obtained for each step of the test defined as *p*_*i*_. The minimum *P* value of all the test was presented as *min*_*i*_(*p*_*i*_). This similar set up works in multivariate analysis. In our situation, a total of 20 genes were selected by the univariate Cox PH model. The set of genes further determined in the multivariate set up to create *min*_*i*_(*p*_*i*_).

**Fig. 1:**
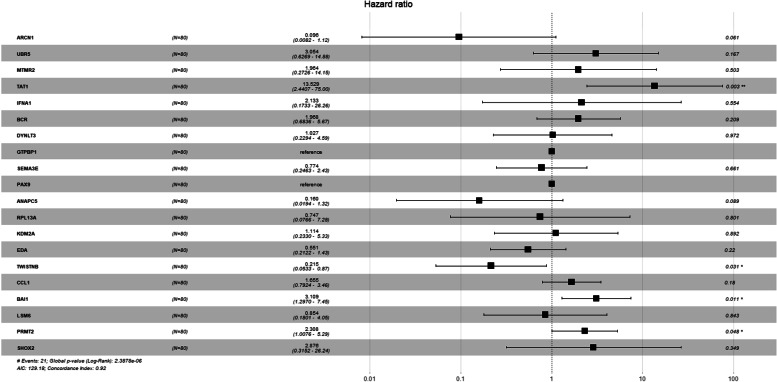
Forest Plot and Chord Plot for overexpressed and underexpressed genes obtained on GSE14764

**Fig. 2:**
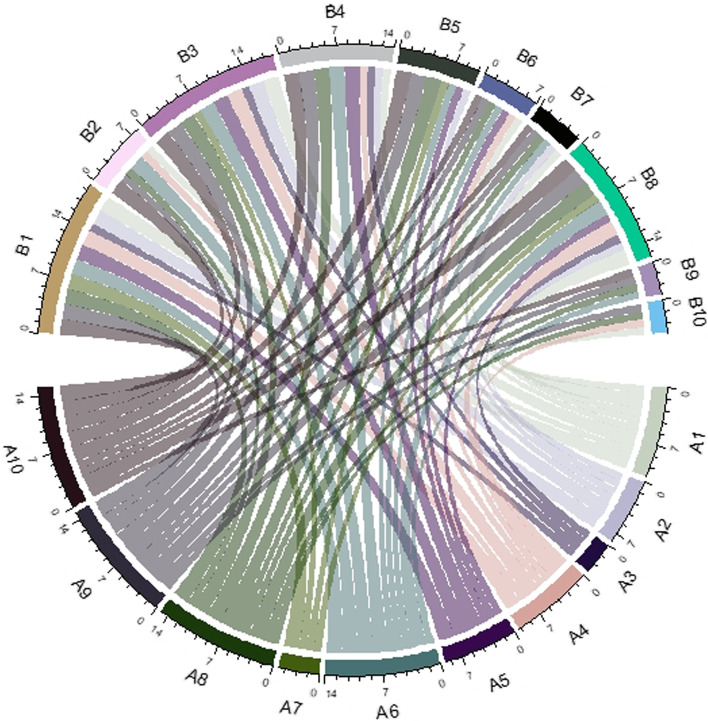
Forest Plot and Chord Plot for overexpressed and underexpressed genes obtained on GSE14764

The *min*_*i*_(*p*_*i*_) was presented as a global *P*-value, which is given in Fig. 1. Global significance level *p*_*min*_ associated with the single experiment-wise statistic was obtained from the proportion of values at least as small as the observed *min*_*i*_(*p*_*i*_) .If *p*_*min*_ <  = 0.05 then the experiment (at least one of the steps in the clustering process) was significant at the 5\% level [25] (Fig. 2).

### Clustering effect

To assess the benefits of clustering, we compared the statistical significance for the entire experiment, which involved the tests at each step created by clustering; that is, we compared the global *p*-value, *p*_*min*_, with the significance level, *p*_0_, of the statistic prior to clustering. When *p*_*min*_ < *p*_0_, the results were more significant, and the clustering was beneficial. But if *p*_*min*_ > *p*_0_ then the smallest *p*-value has a high probability of occurring by chance.

In our study, the ‘ggforest’ function was used with the survminer package for the preparation of the Forest Plot for the Cox PH model. For instance, the Gene Expression Omnibus (GEO) database under the accession number GSE14764 consists of 22,283 probes, and 80 patients and the univariate data analysis were performed. However, all the significant genes showed a non-significant *p*-value. But, the overall *p*-value obtained by the Global *p*-value observed with the highly significant *p*-value.

### Genes links by data visualisation

The circular visualisation method was used to show the relations between genes by links by the chord plots. The “circlize” package in R software was used, and the Chord diagram was formed in a straight forward and highly customised way. For creating the chord diagrams, we first computed a concordance matrix for each dataset. The row and column names of each matrix contained the result as upregulated and down-regulated genes leading to death. Each concordance cell was assigned a value as 0, 1, or 2 by calculating risk towards death due to the respective high values of upregulated and low values of down-regulated genes. Proportions of risk were partitioned as < 25%, more significant than 25 and < 50%, > 50%.

Chord plot showed that the links were straight forward to show the relations between genes (Fig. 2). The width of the links was also observed as proportional to the strength of the relation. The colours of links provided the visual linkage among the genes. The width of sectors represents total strength for the gene, which connects to other gene or was connected from other genes. The forest plot and chord plot for overexpressed and under-expressed genes obtained on GSE14764 consisting of 22,283 probes and 80 patients were done for obtaining the global *p*-value, and similar plots for additional 5 data sets was done.

## Results

As shown in the Table 1, a total of 20 genes from each data sets was selected. With the statistical analysis, we were able to found out the upregulated and down-regulated genes that showed up with the hazard ration (HR) and the *p*-value. In this study, the results obtained describes that the upregulated genes with the HR > 1 are making difficulties for survival and the down-regulated genes with the hazard ratio HR < 1.

**Table 1:** Up Regulated and Down Regulated genes

GSE14764							
Up Regulated genes				Down Regulated genes			
Gene Name	HR	HR Limits	*P*-value	Gene Name	HR	HR Limits	*P*-value
ARCN1	6.4	(2.15,19.07)	0.000	ANAPC5	0.24	(0.11,0.52)	0.000
UBR5	5.32	(2.09,13.51)	0.000	RPL13A	0.26	(0.1,0.68)	0.005
MTMR2	3.19	(1.04,9.79)	0.043	KDM2A	0.4	(0.16,1.01)	0.053
TAT1	3.15	(1.22,8.09)	0.017	EDA	0.44	(0.27,0.7)	0.000
IFNA1	3.08	(1.04,9.15)	0.042	TWISTNB	0.51	(0.27,0.95)	0.033
BCR	2.96	(1.62,5.39)	0.000	CCL1	0.63	(0.44,0.91)	0.012
DYNLT3	2.52	(1.29,4.93)	0.007	BAI1	0.63	(0.46,0.86)	0.003
GTPBP1	2.34	(1.19,4.63)	0.014	LSM6	0.64	(0.45,0.90)	0.011
SEMA3E	1.99	(1.01,3.95)	0.048	PRMT2	0.65	(0.45,0.92)	0.015
PAX9	1.72	(1.06,2.77)	0.027	SHOX2	0.74	(0.55,0.98)	0.034
GSE17260							
Up Regulated genes				Down Regulated genes			
Gene Name	HR	HR Limits	*P*-value	Gene Name	HR	HR Limits	*P*-value
YWHAB	3.24	(1.69,6.2)	0.000	ZNF341	0.24	(0.09,0.64)	0.004
SEC22B	3.21	(1.39,7.43)	0.006	PRR3	0.25	(0.09,0.72)	0.010
EIF3J	3.11	(1.57,6.15)	0.001	TBXA2R	0.25	(0.09,0.69)	0.006
MMP1	3.1	(1.36,7.07)	0.007	OPALIN	0.25	(0.1,0.63)	0.003
BNIP2	3.04	(1.51,6.12)	0.001	SERINC5	0.26	(0.1,0.69)	0.007
FAM126B	2.94	(1.45,5.98)	0.002	AQP10	0.27	(0.1,0.68)	0.005
DTWD1	2.87	(1.5,5.49)	0.001	FAM95B1	0.27	(0.12,0.6)	0.001
CU674465	2.86	(1.33,6.13)	0.007	MIDN	0.33	(0.17,0.64)	0.001
TBC1D15	2.83	(1.45,5.52)	0.002	RABL2A	0.34	(0.17,0.66)	0.001
PHF20	2.7	(1.32,5.53)	0.006	NKPD1	0.34	(0.15,0.77)	0.009
GSE18929							
Up Regulated genes				Down Regulated genes			
Gene Name	HR	HR Limits	*P*-value	Gene Name	HR	HR Limits	*P*-value
Hsp40	11.32	(3.35,38.24)	0.000	ANAPC15	0.09	(0.03,0.32)	0.000
HELZ	8.66	(1.96,38.26)	0.004	MANF	0.11	(0.02,0.55)	0.006
STX3	6.81	(1.74,26.65)	0.005	BAX	0.12	(0.03,0.42)	0.000
FTL	6.32	(1.57,25.5)	0.009	CDC34	0.15	(0.04,0.5)	0.002
NPC1	6.09	(2.28,16.27)	0.000	IL23A	0.15	(0.05,0.44)	0.000
PMM1	5.17	(1.61,16.67)	0.005	NCL	0.16	(0.05,0.49)	0.001
TPP1	4.77	(1.94,11.73)	0.000	PEX10	0.18	(0.06,0.5)	0.001
RBL2	4.27	(1.76,10.34)	0.001	PHGDH	0.19	(0.06,0.53)	0.001
CREG1	4.13	(1.82,9.37)	0.000	DNPH1	0.19	(0.05,0.66)	0.009
AKAP11	3.96	(1.7,9.23)	0.001	SSNA1	0.2	(0.06,0.65)	0.007
GSE30161							
Up Regulated genes				Down Regulated genes			
Gene Name	HR	HR Limits	*P*-value	Gene Name	HR	HR Limits	*P*-value
CDHR3	6.63	(1.95,22.52)	0.002	WNT16	0.04	(0.01,0.24)	0.000
STK25	5.92	(2.18,16.07)	0.000	CYP2E1	0.09	(0.03,0.34)	0.000
NADK2	5.28	(2.18,12.78)	0.000	CAPN3	0.1	(0.04,0.26)	0.000
HELQ	5.07	(1.66,15.46)	0.004	EP400	0.11	(0.03,0.37)	0.000
USF3	4.41	(1.53,12.7)	0.005	CIB2	0.11	(0.03,0.41)	0.001
HKR1	4.37	(1.52,12.58)	0.006	DUSP4	0.13	(0.03,0.56)	0.006
MAGOH	4.3	(1.83,10.12)	0.000	ZDHHC2	0.14	(0.04,0.44)	0.000
TLE4	3.91	(1.46,10.5)	0.006	CCT6B	0.14	(0.04,0.45)	0.000
TRIO	3.84	(1.49,9.86)	0.005	LOC101929607	0.14	(0.03,0.59)	0.007
POSTN	3.76	(1.55,9.11)	0.003	FOXJ1	0.14	(0.06,0.37)	0.000
GSE49997							
Up Regulated genes				Down Regulated genes			
Gene Name	HR	HR Limits	*P*-value	Gene Name	HR	HR Limits	*P*-value
LTBP2	1.79	(1.37,2.35)	0.000	COL16A1	0.39	(0.19,0.77)	0.007
ACTA2	1.79	(1.31,2.44)	0.000	RFX4	0.47	(0.31,0.71)	0.000
WBP4	1.77	(1.21,2.59)	0.003	RPP38	0.5	(0.32,0.76)	0.001
LOC283241	1.71	(1.12,2.63)	0.013	BCR	0.55	(0.37,0.83)	0.004
CYB561D2	1.7	(1.22,2.38)	0.001	ADRA1D	0.57	(0.38,0.86)	0.006
CTSK	1.67	(1.18,2.36)	0.003	TTN	0.57	(0.4,0.82)	0.002
NAP1L5	1.63	(1.19,2.25)	0.002	ZEB2	0.58	(0.4,0.84)	0.004
DCN	1.63	(1.23,2.17)	0.000	CDH1	0.58	(0.38,0.89)	0.012
ZBTB7	1.6	(1.19,2.16)	0.001	KLC2	0.59	(0.41,0.86)	0.006
KIF1A	1.6	(1.12,2.29)	0.009	FAP	0.6	(0.37,0.96)	0.033
GSE63885							
Up Regulated genes				Down Regulated genes			
Gene Name	HR	HR Limits	*P*-value	Gene Name	HR	HR Limits	*P*-value
OR7C1	15.43	(3.74,63.59)	0.000	DGCR8	0.1	(0.03,0.28)	0.000
ZSWIM1	13.45	(3.62,50.01)	0.000	MMP1	0.17	(0.07,0.42)	0.000
PITPNA	7.36	(2.09,25.89)	0.001	GOLGA8G	0.22	(0.08,0.61)	0.003
DLL3	7.25	(1.76,29.79)	0.006	CCNE1	0.23	(0.07,0.78)	0.018
LOC92249	6.69	(2.58,17.35)	0.000	ADK	0.23	(0.09,0.59)	0.002
CLASP1	5.97	(1.64,21.74)	0.006	BCL2L12	0.23	(0.08,0.66)	0.006
MBNL1	5.79	(1.04,32.09)	0.044	LOC149478	0.24	(0.1,0.57)	0.001
GP2	4.93	(1.25,19.53)	0.023	PTPN2	0.27	(0.14,0.52)	0.000
C19orf20	4.89	(1.21,19.73)	0.025	SRP72	0.29	(0.16,0.53)	0.000

The results obtained on GSE14764, the genes obtained were ARCN1, UBR5, MTMR2, TAT1, IFNA1, BCR, DYNLT3, GTPBP1, SEMA3E and PAX9, which were upregulated genes and ANAPC5, RPL13A, KDM2A, EDA, TWISTNB, CCL1, BAI1, LSM6, PRMT2 and SHOX2 were down-regulated genes. The genes were ranked based on their HR and Confidence Intervals. The expression of SEMA3 family members was frequently associated with overall patient survival. SEMA3E primarily associated with a poor prognosis of survival. Results reveal an undiscovered role of SEMA3E in promoting pancreatic cancer pathogenesis, suggesting that SEMA3E as a suitable prognostic marker and therapeutic target for pancreatic cancer [26]. PAX9 an independent prognostic factor for the surgical treatment of ESCC and a possible predictor of radiation sensitivity [27]. MTMR2 an essential promoter in gastric cancer invasion and metastasis by inactivating IFNA1/STAT1 signalling and acts as a new prognostic indicator and a potential therapeutic target for gastric cancer [28].

UBR5 a key regulator of cell signalling relevant to broad areas of cancer biology [29]. DYNLT3 exerts pro-tumoral effects on Ovarian cancer through promoting cell proliferation, migration and invasion. DYNLT3 a potential prognostic predictor in ovarian cancer [30]. GTPBP1, a regulator and adaptor of the exosome-mediated mRNA turnover pathway [31]. CCL1 significantly correlated with the infiltration of immunosuppressive FoxP3+ Treg that were known to negatively affect survival negatively. Thus, CCL1 serves as a prognostic marker and novel therapeutic target in breast cancer [32]. High KDM2A levels were correlated with poor prognosis in NSCLC patients. They were suggesting that KDM2A may be a promising therapeutic target in NSCLC [33]. PR (PRDI-BFI and RIZ) domain-containing (PRDM) proteins have been shown to be important in several types of human cancer [34]. RPL13A, the most suitable reference gene for analysing the transcription profile of ovarian cancer cells following treatment with PTX and HCPT [35]. Levels of BAI1 mRNA steadily downregulated in cells lines, primary glioma specimens and from lung adenocarcinoma in brain metastases [36]. PRMT2 were significantly high in malignant breast tissues than in normal tissues of breast [37].

Genes under data set GSE17260 were grouped YWHAB, SEC22B, EIF3J, MMP1, BNIP2, FAM126B, DTWD1, CU674465, TBC1D15 and PHF20 as upregulated, and down-regulated genes were ZNF341, PRR3, TBXA2R, OPALIN, SERINC5, AQP10, FAM95B1, MIDN, RABL2A and NKPD1. The gene YWHAE was found associated with tumour size, lymph node metastasis, and poor patient survival in patients with breast cancer [38]. SEC22B was observed closely related to tumorigenesis with types of mutation. The gene fusion of SEC22B confirmed in aggressive breast cancers and mantle cell lymphoma [39]. EIF3J-AS1 gene was found correlated with prognostic features, including tumour size, vascular invasion and tumour stage, which takes crucial expert roles in hepatocellular carcinoma (HCC) progression [40]. High MMP1 expression associates with worse OS in breast cancer patients after systematic therapy [41].

In the case of Cancer epigenetics, DTWD1 was down-regulated in gastric cancer cell lines and primary gastric carcinoma tissues. DTWD1 functions as a tumour suppressor play an important role in the pathogenesis of many cancers, including gastric cancer [42]. The stimulator of IFN genes mediated DNA sensing pathway plays an important role in the innate immune response to pathogen infection, autoimmunity, and cancer, which was regulated by TBC1D15, mitochondrial dynamics mediators [43]. Plant homeodomain finger protein 20 (PHF20) was highly expressed in primary human gliomas, and its expression was found to be associated with tumour grade, which relates to glioblastoma [44]. There was an increase in overall cancer incidence among patients with primary immunodeficiencies of ZNF341 [45]. Thromboxane synthases were differentially expressed in human breast cancer. TBXA2R thus has a significant prognostic value in clinical breast cancer [46]. FAM95B1 significantly correlates with cervical lymph node metastasis. Cervical lymph node metastasis was an important prognostic indicator for papillary thyroid carcinoma (PTC) and affects treatment strategies (https://www.ncbi.nlm.nih.gov/pmc/articles/PMC6388952/) and tumour staging.

On analysis of GSE18929, genes such as Hsp40, HELZ, STX3, FTL, NPC1, PMM1, TPP1, RBL2, CREG1 and AKAP11 were upregulated genes, and the down-regulated genes were ANAPC15, MANF, BAX, CDC34, IL23A, NCL, PEX10, PHGDH, DNPH1 and SSNA1. Hsp40 was the probes in cancerous lung tissues; it was also shown that levels of Hsp40 increased in the serum of cancer patients [47]. The roles of Syntaxin 3(STX3) acts as an oncogenic protein in human breast cancer [48]. Ferritin Light Chain (FTL) completes with long noncoding RNA to regulate chemoresistance and metastasis of colorectal cancer, which was a leading cause of cancer deaths [49]. NPC1 family of proteins plays an essential role in molecular mechanisms in breast cancer cells which was associated with constitutive activation of autophagy [50]. Expressed gene PMM1, located on chromosome bands, helps differentiate gene expression before and after radiation of subcutaneous fibroblasts, identifying breast cancer patients resistant to radiation-induced fibrosis [51]. A rare variant of TPP1 confers an increased risk of colorectal cancer through interrupting TPP1-TIN2 interaction [52]. RBL2/p130, a member of the retinoblastoma family of proteins, a well-known tumour suppressor gene in the Rb family, found inactivated in numerous cancers, has growth-suppressive properties and also deregulates in various types of cancer, especially in Pancreatic adenocarcinoma (PDAC), one of the most aggressive malignancies in humans [53].

CREG1, the cellular repressor of E1A-stimulated genes, a downstream effector of KRAS which identifies in Glycoproteomic Approach as a positive regulator of CREG1 in Non-small Cell Lung Cancer Cells [54]. The AKAP gene was related to tumour heterogeneity in breast cancer tumours which relates to the primary tumour [55]. The adenomatous polyposis coli (APC) play a rate-limiting role in the majority of sporadic colorectal cancers. Loss of APC function triggers the chain of molecular and histological changes [56]. MANF levels were associated with the status of liver cirrhosis, advanced tumour-node-metastasis (TNM) stage, and tumour size [57]. Frameshift mutations were seen present in both BAX alleles in some MMP+ colon tumour cell lines and in primary tumours. Inactivating BAX mutations during the progression of colorectal MMP+ tumours and the wild-type BAX gene plays a suppressor role in a p53-independent pathway for colorectal carcinogenesis [58]. CDC34 changes expressions of Proteasome and Ubiquitin Genes in Human Renal Cancer Cells (https://cancerres.aacrjournals.org/content/51/24/6677.short). NCL was commonly overexpressed in human breast tumours, and it was expression correlates with NCL dependent miRNAs [59]. Phosphoglycerate dehydrogenase (PHGDH) plays an essential role in cancer-specific metabolic reprogramming [60]. The crystal structure of rat DNPH1, a potential target for anti-cancer therapies, suggested that various analogues of AMP can inhibit this enzyme [61].

Results obtained on GSE30161 were grouped CDHR3, STK25, NADK2, HELQ, USF3, HKR1, MAGOH, TLE4, TRIO and POSTN as upregulated, and the down-regulated genes were WNT16, CYP2E1, CAPN3, EP400, CIB2, DUSP4, ZDHHC2, CCT6B, LOC101929607 and FOXJ1. The glutamine-dependent survival and sensitivity to ER stress in USF3-deficient cells provided adjunct preventive interventions for both sporadic cancers as well as cancer predisposition syndromes [62]. HKR1 mRNA expression levels in lung cancers were higher, and that high expression levels in lung cancers were found to be associated with antemortem platinum drug administration [63]. MAGOHB, the top gene dependency in cells with hemizygous MAGOH deletion. MAGOH and MAGOHB as reciprocal paralog dependencies across cancer types suggest a rationale for targeting the MAGOHB-IPO13 axis in cancers [64]. TLE4 promote colorectal cancer progression through activation of the signalling pathway. TLE4 in colorectal cancer (CRC) tissues were significantly higher than that in their matched adjacent intestine epithelial tissues. TRIO gene promotes Colorectal Cancer Invasion and Metastasis. TRIO pY2681, one of the downstream effectors in colorectal cancer and can be a prognostic marker, helping to determine the therapeutic modality of patients with colorectal cancer [65].

The vascular endothelial growth factor was found to be a significant regulator of breast cancer angiogenesis, the effects of which were transmitted through the kinase domain receptor (KDR). Up-regulation of KDR by periostin (POSTN) induces angiogenesis which was an important step in the development of cancer [66]. Gastric cancer was a multi-step, multi-factor, and elaborated process that was associated with gene abnormal gene expression. Great significant modification occurred in tumoral tissues, and the gene expression increased significantly in tumoral tissue observed due to upregulation of WNTt16 gene expression in gastric cancer, which was one of the most severe and lethal kinds of cancer in the world [67]. Investigation of the association between cancer development risk and cytochrome P4502E1 (CYP2E1) gene polymorphism was significant [68]. A novel fusion gene, EP400-PHF1, was discovered in ossifying fibromyxoid tumour; its relation to this type of tumour has been uncertain because the EP400-PHF1 fusion gene has been successfully detected in only 1 case [69]. CIB2, significantly down-regulated in ovarian cancer, and low CIB2 expression was associated with poor prognosis in ovarian cancer patients [70]. Dual-specificity protein phosphatase 4 (DUSP4), a negative regulator of extracellular-regulated kinase activity, was a potential mediator of resistance to chemotherapy and a tumour suppressor. Clarification was done for the association between DUSP4 gene expression and clinical outcome in patients with colorectal cancer [71]. Zinc finger, DHHC-type containing 2 (ZDHHC2), proposed as a putative tumour/metastasis suppressor gene and was often aberrantly decreased in human cancers. ZDHHC2 expression pattern and its clinical significance have not yet been investigated in gastric adenocarcinoma [72]. Forkhead box (FOX) proteins were a large family of transcriptional regulators, which control a variety of biological processes leading to alteration of cell fate. Thus, the development and progression of ovarian cancer, which was the most lethal of all gynaecological malignancies, and the Identification of novel prognostic and therapeutic targets for ovarian cancer was crucial. As four FOX proteins, including FOXO1, FOXO3a, FOXJ1 and FOXB1, were the likely targets of NANOG in embryonic stem cells [73].

On thorough analysis of GSE49997, we found LTBP2, ACTA2, WBP4, LOC283241, CYB561D2, CTSK, NAP1L5, DCN, ZBTB7 KIF1A were the upregulated genes, and down-regulated genes were COL16A1, RFX4, RPP38, BCR, ADRA1D, TTN, ZEB2, CDH1, KLC2 and FAP. The resulting genes were not exceptional as they have been previously termed as less or more effective in cancer oncology. The genes which were being termed as an essential marker for significant prognosis of cervical cancer were ADRA1D, LTBP2 whereas KLC2 protein-protein was termed for poor prognosis in early NsCLC patients [74]. RFX4 was considered as a target for GMB treatment. As a result, it depicts to be a risk factor for stemness of GSCs and malignance of Flioma. On comparing the lower express ZBTB7 with the higher, which exhibited lower overall and recurrence-free survival, hence ZBTB7 may be necessary for the initiation and progression of TCC (Urothelial carcinoma, also known as transitional cell carcinoma) [75]. ACTA2 can be considered as a prognostic biomarker and therapeutic target for metastatic lung cancer [76]. CTSK, in the case of ovarian cancer, shows association with metastases and inferior overall prognosis of EOC (Epithelial ovarian cancer) [77].

NAP1L1 was overexpressed while promoting the proliferation of p57 promoter Methylation. Cervical cancer has been seen initiated and progressed by an oncogene COL6A1. It was leading to a poor prognosis of cervical cancer [78]. TTN-AS1 paves a path for new treatment strategies in Cervical cancer patients, and it shows a significant correlation with FIGO stage, poor differentiation, lymph node metastasis and poor overall survival of CC patients [79]. Upregulated Zeb2 has an association with the progression of cancer. It was found to be relatively higher in colon cancer cell lines but seen reduced in healthy human colonic epithelial cell lines [80]. CDH1 shows the correlation with cervical cancer carcinogenesis as well as histological subtypes [81]. HGSOC, the most common and lethal form of ovarian cancer, Upregulation of FAP was found in advanced stage HGSOC patients, showing association with poor prognosis via FN1 pathway, the association of FAP network shows FN1 can be a potential downstream gene leading to HGSOC survival [82]. Whereas downregulation of STK25 triggers a mechanism by which tumour cells functionally impair the hippo tumour tumour-suppressor pathway [83].

Results obtained on GSE63885 were grouped OR7C1, ZSWIM1, PITPNA, DLL3, LOC92249, CLASP1, MBNL1, C21orf84, GP2 and C19orf20 as upregulated genes and downregulated genes were DGCR8, MMP1, GOLGA8G, CCNE1, ADK, BCL2L12, LOC149478, SPPL2B, PTPN2, SRP72. The genes were ranked based on their HR and Confidence Intervals.OR7C1, a novel marker for colon CICs and a target of potent CIC-targeting immunotherapy [84]. `The activation sensitive nature of ZSWIM1 expression shows that it plays a novel role in the development or function of T helper cells, which primarily mediate anti-tumour immunity [85]. A therapeutic strategy for the treatment of cancer could be idealised on targeting the PITPNA-AS1-associated signalling, which mediates the effects of c-MET on the proliferation, apoptosis and cell cycle in cervical cancer cells [86]. DLL3 localised to the plasma membrane of tumour cells and acted as a reliable biomarker to predict cancer progression and a poor clinical outcome. Pre- and clinical trial results indicated that membrane DLL3, a potential target for preventing tumour growth [87].

Endothelial cell (EC) branching was critically dependent upon the dynamic nature of the microtubule (MT) cytoskeleton. CLASP1 knockdown results were significantly faster and longer-lived MT growth specifically within EC branches, and thereby identify CLASP1 as a critical regulator of MT dynamics within EC branches. MLL-rearranged signatures revealed that muscleblind-like 1 (MBNL1) was one of the most consistently overexpressed genes in MLL-rearranged leukaemia compared to other leukaemias [88]. Mediated by miR-27b, DGCR8 functions as oncogene in Ovarian cancer [89]. MMP-1, differentially regulated in breast cancer tissues and served a role in breast cancer invasion and metastasis. Hence, it was considered as a diagnostic marker and drug target for breast cancer [90]. CCNE1 gene was targeted by miR-16-1 in Cervical Cancer cells [91]. BCL2L12 expression and stimulated proliferation and engrafting of leukaemia cells suggested CD82 and BCL2L12 as promising therapeutic targets in AML [92]. It was anticipated that the absence of SPPL2a/b critically affects disease-relevant pathways in the brain but also other organ systems when mice were challenged in a certain way [93]. PTPN21 overexpression was an early step in urothelial cancer progression. It was a novel biomarker and possible therapeutic target for bladder cancer [94]. SRP72, a novel gene involved in radio resistance [95].

## Discussion

As of now, we have seen that ovarian cancer is the most common gynaecological oncological malignant tumour. It is the leading factor for the cause of death among women worldwide due to its late diagnosis and poor prognosis. Like any other cancer, it exhibits complexity and heterogeneity drug response and overall survival. Our investigation for the gene expression for ovarian cancer reveals that there were a number of upregulated and downregulated genes that were useful in disease diagnosis for an individual, as we have already mentioned about them earlier.

Ovarian carcinoma contributed to the highest mortality rate for any kind of gynaecological malignancies. It was investigated that the molecular markers can predict the death outcome of ovarian cancer independently along with clinical predictors [19]. In a study [20], it was shown that 110 patients from the Japanese population had advanced-stage serous ovarian cancer, where 93(84.5%) patients were in stage-III and rested in stage-IV. After having primary surgery, all the patients were treated with platinum/ taxane-based chemotherapy. The median duration of overall survival was found to be 31 months, and the total of 88-gene expression profile was found significant by ridge Cox proportional model. The clinical factors were explored in predicting cancer progression [20]. Furthermore, Jazaeri and Konstantinopolous in their studies showed about the gene expression profile of BRCAness, which was prepared from the publicly available microarray data set that included tumour expression data from 61 patients with pathologically confirmed EOC, including 34 with BRCA germline mutations (*n* = 18, BRCA1; *n* = 16, BRCA2), and 27 without either mutation (i.e., sporadic cancers). The hierarchical clustering was used to define the BRCA-like (BL) and non-BRCA-like (NBL)). Similar to our study, Ferris(2012) attempted the multi-gene molecular predictors to forecast the response of 55 ovarian cancer patients. They studied the overall survival and multi-gene molecular predictor. The predicted responders and the non-responders with a median survival of 55.4 months vs 32.1 months respectively showed a significant difference which was demonstrated by the combination predictors. Finally, it was concluded that the COXEN single and the combination was a drug predictor that successfully stratified the platinum resistance and taxane response in ovarian cancer [22].

In our study, we performed the overall survival and death as an outcome of interest to select the genes, whereas, in a study performed by Pils (2012), it was given that the RNA from the fresh frozen tumour was separated by ABI PRISM 6100 Nucleic Acid PrepStation (Applied Biosystems, Carlsbad, CA, USA). The correlation analysis of the clinicopathologic parameters was examined through chi-square test, t-test and Fisher’s exact test where the progression-free survival and overall survival; were measured. And, a total of 194 ovarian cancer patient’s clinic pathological and microarray data was selected to select the influencing genes on disease survival [23].

## Conclusions

Ovarian cancer is the leading cause and adding to the disease burden all over the world. We discovered the expression of the number of genes to understand the effectiveness or harmfulness of those upregulated and down-regulated genes for the better prognosis of cancer. With the use of integrative data analysis, insight on the gene to make the influence of better survival was done. The results obtained with individual studies show little replicability, even with similar clinical outcomes. Study design and small sample size are critical limitations to deal with heterogeneity. The individual study may lead to low sensitivity because of the small sample size. The performance of sensitivity steadily decreases with an increase in heterogeneity of a gene effect. Besides, it was possible to combine multiple studies and improvise variable selection. It leads to higher prediction power by adopting power from different studies.

This study shows how to combine the effects of the same variable from the studies and make an impact on clinical prediction. The gene signature variability makes impacts on prediction. Besides, it was possible to combine the positive and negative genes and convert them into an adjacency matrix to prepare the chord plot, and that after processing the variable selection. Data obtained from multiple sources are subject to additional processing to obtain it in an equal platform. Finally, we provide applications on public domain data to select the gene signature. The achievement of the gene signature in the chord plot relies upon the over and under-expressed genes together.

## Data Availability

Data is publicly available.
